# One-Year Efficacy and Safety of Combined Photorefractive Keratectomy and Accelerated Corneal Collagen Cross-Linking after Intacs SK Intracorneal Ring Segment Implantation in Moderate Keratoconus

**DOI:** 10.1155/2019/7850216

**Published:** 2019-07-08

**Authors:** Il Hwan Koh, Kyoung Yul Seo, Seong Bae Park, Hun Yang, InSik Kim, Jin Sun Kim, David G. Hwang, Sang Min Nam

**Affiliations:** ^1^SU Yonsei Eye Clinic, Seoul, Republic of Korea; ^2^Department of Ophthalmology, Institute of Vision Research, Eye and Ear Hospital, Severance Hospital, Yonsei University College of Medicine, Seoul, Republic of Korea; ^3^Cornea Service and Refractive Surgery Service, Department of Ophthalmology, University of California, San Francisco, San Francisco, CA, USA; ^4^Department of Ophthalmology, CHA Bundang Medical Center, CHA University, Seongnam, Republic of Korea

## Abstract

**Purpose:**

To report one-year outcomes of a modified version of two-stage multimodal surgical protocol for moderate keratoconus which has been suggesting promising preliminary results.

**Materials and Methods:**

30 eyes of 25 patients with moderate keratoconus who exhibited visual complaints and/or disease progression were included for this retrospective case study. Approximately 3 months after implantation of intracorneal ring segment (Intacs SK™), a combination of corneal wavefront-guided transepithelial photorefractive keratectomy (CWG-transPRK, Schwind Amaris® 1050, and Schwind Sirius) and accelerated collagen cross-linking (accCXL, Avedro KXL™) was performed. Patients were examined for uncorrected and corrected distance visual acuity (UDVA; CDVA), keratometric power (K), corneal thickness, and corneal higher-order aberrations (HOAs) preoperatively and at postoperative 1, 3, 6, and 12 months.

**Results:**

The median UDVA and mean CDVA were enhanced from 6/38 to 6/12 and from 6/19 to 6/7.5, respectively, through 12 months after CWG-transPRK/accCXL. The 12-month CDVA of all patients was better than 6/12 Snellen, and no subject lost one or more lines of CDVA. The magnitudes of both myopia and corneal steepness were decreased in turn by Intacs SK implantation and also by CWG-transPRK/accCXL, but the reduction in HOA was largely the result of CWG-transPRK/accCXL. The magnitude of corneal thinning stabilized within 3 months after CWG-transPRK/accCXL.

**Conclusion:**

This approach may allow patients with moderate keratoconus to obtain satisfactory vision without the need for contact lens wear. This surgery appeared to be effective and safe through 1 year of follow-up.

## 1. Introduction

Keratoconus is a noninflammatory ectatic disorder that, depending on the magnitude of steepening and irregular astigmatism resulting from its progressive phase, can result in varying magnitudes of visual acuity deterioration [1]. Recently, surgical procedures such as intrastromal corneal ring segment (ICRS) implantation, photorefractive keratectomy (PRK), and corneal collagen crosslinking (CXL) have become available as treatment considerations [2, 3]; however, each of these procedures, on a standalone basis, has limitations in terms of efficacy, stability, predictability, and safety.

ICRS implantation in moderate keratoconus can improve vision by reducing refractive error and corneal curvature, stabilizing corneal shape, and increasing tolerance for contact lenses [4, 5]. ICRS shows the same successful rate in different elevation or corneal topography patterns [6]. Notwithstanding, the refractive effects of an ICRS as a standalone procedure may be unpredictable, leading to disappointing outcomes particularly for patients with mild to moderate keratoconus who may have good vision preoperatively [5]. In addition, the ability of an ICRS to halt keratoconus progression has not been consistently demonstrated [4, 5], and residual irregular astigmatism or other complications can lead to patient dissatisfaction [7, 8].

Topography-guided PRK can address myopia and corneal irregularities, but on a standalone basis, the magnitude of correction required may compound thinning in an already ectatic cornea, risking further progression of the keratoconus [11, 12]. Cross-linking (CXL) can prevent progression but has minimal effect on reducing steepening, myopia, and irregular astigmatism [2, 13]. Simultaneous PRK and CXL can improve the refractive status but a high rate of postoperative progression of keratoconus is observed [14, 15].

Accordingly, a multimodal approach may serve to combine the desirable attributes of each of the three procedures while minimizing the individual limitations associated with each [9, 10, 13, 16, 17]. Al-Tuwairqi and colleagues described ICRS implantation using the Keraring (Mediphacos, Brazil) followed by simultaneous topography-guided transepithelial PRK combined with conventional CXL [9]. Lee* et al*. combined Keraring implantation followed later by simultaneous corneal wavefront-guided transepithelial PRK (CWG-transPRK) and accelerated CXL (accCXL) [10].

The current study employed CWG-transPRK approach with accCXL but proposed three modifications: the use of a corneal topographer (Schwind Sirius topographer, SCHWIND eye-tech-solutions GmbH, Kleinostheim, Germany) that employed both Placid-based data and Scheimpflug-based tomography data to increase the accuracy of aberration correction [18] and the use of Intacs SK (Addition Technology Inc., Lombard, IL, USA) to provide a potentially larger effective zone of flattening of the cone [19]. In addition, the current study chose a protocol with a shorter time for ultraviolet irradiation of 3 minutes at 30 mW/cm^2^ rather than the 6 minutes at 15 mW/cm^2^ as was used by Lee and coauthors [10].

Finally, the current study lengthened the follow-up time to 1 year compared to the 6-month follow-up period of the prior studies [10]. Unlike the previous Lee report, this study also performed a safety analysis of lines loss of CDVA [10].

## 2. Materials and Methods

Records were reviewed of all patients who underwent two-stage surgery to treat keratoconus at SU Yonsei Eye Clinic (Seoul, Korea) between 2015 and 2016. By the single physician (IH Koh), all surgeries were performed, and the patient was followed up using a uniform protocol. Preoperative and postoperative uncorrected distance visual acuity (UDVA) and CDVA, manifest refraction, autorefraction, mean keratometric power (K), and steep K were recorded. In addition, the maximum K and thinnest corneal thickness (CT_thinnest_) were measured using an Oculyzer II (Alcon, Fort Worth, TX), and corneal higher order aberrations (HOAs) were obtained using the Schwind Sirius. Postoperative measurements were recorded at the times of ICRS implantation and the second surgery and at 1, 3, 6, and 12 months later.

Keratoconus was diagnosed on the basis of corneal tomography and clinical findings on slit lamp examination [2, 3]. The included patients are those who complained of visual problems with the use of glasses and/or exhibited keratoconus progression over 6 months. Progression was defined as one or more of the following changes: an increase ≥ 1.0 D in the maximum K or the manifest cylinder or a decrease in visual acuity ≥ 1 line without any other etiology. No patient was pregnant and had severe corneal scarring, a history of herpetic keratitis, or a systemic autoimmune disease. The thinnest corneal thickness was no less than 440 *μ*m before the PRK. Institutional Review Board (CHA Bundang Medical Center, CHA University, Seongnam, Republic of Korea) approval was obtained.

### 2.1. Surgical Technique

In the first step, an Intacs SK intracorneal implant (6.0 mm optical zone; fixed arc length of 150°; angulation 30°) was implanted in the cornea. The ring segment thickness was decided according to the nomogram of the manufacturer [19]. If the topographic steep meridian passed through the apex of the cone and divided the ectatic area into approximately equal halves, symmetric segments were used. Otherwise, asymmetric segments were implanted.

Each channel for Intacs SK placement was created using a VisuMax femtosecond laser (Carl Zeiss Meditec, Dublin, CA, USA) with a pulse energy of 300 nJ. The depth of the ring channel was set to 75–80% of the thinnest corneal thickness of the chosen tunnel. The inner and outer tunnel diameters were preset to 5.8–5.95 mm and 7.05–7.2 mm, respectively, depending on the K reading and ring thickness. In addition, a 1.35 mm radial entry incision was created. After implantation of the Intacs SK, one drop of 0.5% (w/v) moxifloxacin (Vigamox; Alcon, Fort Worth, TX, USA) was applied and a bandage contact lens was placed on the cornea. The lens was removed the next day and topical antibiotics with steroids were applied four times daily for 2 weeks.

Patients were followed up on postoperative day 1, week 1, week 2, and week 4 and then every 1 month. The second step of the surgery was performed if the mean K did not decrease by more than 1 diopter from the previous K, typically at 3 months after ICRS implantation. However, if the mean K increased above the previous K by 1 D or more at 4 weeks or more after surgery, the second step was proceeded not to lose the flattening effect of ICRS. CWG-transPRK was executed using the transepithelial mode of the Schwind Amaris 1050 RS excimer laser (SCHWIND eye-tech-solutions GmbH, Kleinostheim, Germany). Employing the ORK-CAM module, an aspheric ablation profile was created prior to refractive laser treatment based on corneal wavefront data. The Schwind Sirius (a combination of a rotating Scheimpflug camera and a small-angle Placido disk topographer with 22 rings) produced a series of 25 Scheimpflug images and one Placido top-view image [18]. First, HOAs of the anterior corneal surface were corrected and refractive errors were partially rectified, but only when the corneal thickness was adequate [20]. If full HOA correction was difficult because of corneal thinning, the minimal depth mode of the ORK-CAM module was used for partial correction of the HOAs. To avoid iatrogenic ectasia, the maximum ablation depth was limited to 60 *μ*m in the area of the keratoconus cone. Mitomycin C (0.02% w/v) was applied for 20 seconds immediately after CWG-transPRK to prevent the development of haze with regression. Shortly after ablation was completed, accelerated CXL was performed. VibeX Rapid (Avedro Inc., Waltham, MA; 0.1% [w/v] riboflavin in hydroxypropyl methylcellulose) was instilled in the operative eye every 2 minutes for 10 minutes. Next, the corneal surface was thoroughly rinsed with a sterile balanced saline solution. If the residual thickness was measured ≤ 400 *μ*m by ultrasound pachymetry, normal saline or distilled water was repeatedly added to make the cornea swell. The cornea was exposed to ultraviolet A light at a wavelength of 365 nm for 3 minutes at an irradiance of 30 mW/cm^2^, to give a total radiant exposure of 5.4 J/cm^2^, using the KXL System (Avedro). After accelerated CXL, the corneal surface was irrigated with cold balanced salt solution and a bandage contact lens was applied. Levofloxacin 0.5% (w/v) (Cravit; Santen, Osaka, Japan) was applied four times daily for 1 week. Fluorometholone (0.1%, w/v) was applied twice daily for 8 weeks and then tapered over 4–6 weeks.

### 2.2. Statistics and Calculations

The* safety index* was calculated as the ratio of the postoperative best corrected distance visual acuity (CDVA, decimal) to the preoperative CDVA. The* efficacy index* was the ratio of postoperative uncorrected distance visual acuity (UDVA, decimal) to the preoperative CDVA. Corneal higher-order aberrations (HOA) were measured at the 5 mm zone of the cornea. The total HOA RMS was computed for the third-to-seventh Zernike terms. Prism 7 for Mac OS X (version 7.0a, GraphPad Software Inc., La Jolla, CA), Microsoft Excel for Mac 2011 (version 14.5.2; Microsoft, Inc., Redmond, WA), and MedCalc (version 12.7.7.0; MedCalc Software, Ostend, Belgium) were used for statistical calculations and graphical analyses.

## 3. Results

A total of 30 eyes from 25 patients met the study enrollment criteria, and the data obtained on these patients through one year of follow-up were analyzed. Medians rather than means were selected as a measure of centrality when the distribution was highly skewed by outliers, such as with the distribution of preoperative UDVA, which included a few highly myopic eyes.

The mean patient age was 27 ± 6 (SD) years (range, 19–47 years), and the male:female ratio was 17:8. The preoperative measurements were displayed on Table 1. In addition, the medians of spherical equivalent (SE) and absolute cylinder values derived by autorefraction were -7.8 D (range, -21.9 D to -2.5 D) and 5.6 D (range, 2.0 D - 12.0 D), respectively. As a result, the average keratoconus grade was stage 2 according to the modified Krumeich classification (Table 1) [3]. By comparison, the keratoconus severity in the study by Al-Tuwairqi et al. [9] was stage 1 and that of Lee et al. was borderline stage 2 (Table 1) [10].

The mean interval between ICRS implantation and PRK-crosslinking surgery (PRK-CXL) was 92 ± 54 (SD) days (range, 29-226 days). For PRK-CXL, the optical zone ranged from 6.0 to 6.6 mm and the average maximum ablation depth was 67.62 ± 26.52 (SD) *μ*m.

**Figure 1:**
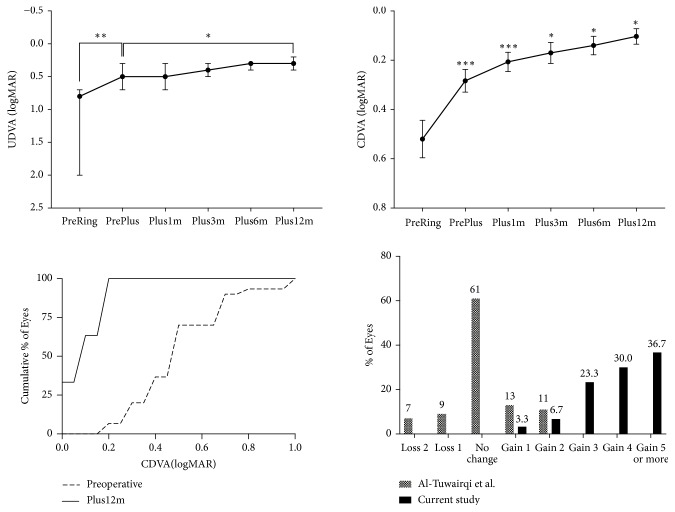
*Visual outcomes of the two-staged multimodal surgery for keratoconus.* The error bars (top left and right graphs) are the 95% confidence intervals of the median (UDVA) or the mean (CDVA). Cumulative distribution (bottom left graph) was significantly changed after surgery (*P* < 0.001, Kolmogorov-Smirnov test). Changes in lines of CDVA (bottom right graph) are compared between the report of Al-Tuwairqi et al. [9] and current study; CDVA changes were not reported by Lee et al. [10] and thus could not be shown. UDVA, uncorrected distant visual acuity; CDVA, corrected distant visual acuity; PreRing, before intracorneal ring segment implantation; PrePlus, before PRK-CXL (“Plus” surgery); Plus1m, 1 month after PRK-CXL; Plus3m, 3 months after PRK-CXL; Plus6m, 6 months after PRK-CXL; Plus12m, 12 months after PRK-CXL. *∗∗∗*,* P* < 0.001; *∗∗*,* P* < 0.01; *∗*,* P* < 0.05; change from the previous value; other pairs are indicated with lines (Friedman test and Dunn's multiple comparisons test for UDVA; repeated ANOVA and Sidak's multiple comparisons test for CDVA).

CDVA improved after the combination of ICRS implantation followed later by PRK-CXL (Figure 1). The mean CDVA was 0.3 ± 0.1 (SD) logMAR (6/12 Snellen) after intracorneal ring implantation and improved to 0.1 ± 0.1 (SD) logMAR (6/7.5 Snellen) at 12 months after PRK-CXL. The 20% of eyes had preoperative CDVA better than 6/12 Snellen (0.3 logMAR), but the 100% of eyes had CDVA higher than 6/12 Snellen at 12 months after the second stage of the surgical protocol (Figure 1). UDVA was enhanced after each stage of treatment: the median of UDVA was enhanced after ICRS implantation to 0.5 logMAR (6/19 Snellen) and further improved to 0.3 logMAR (6/12 Snellen) at 12 months after PRK-CXL (Figure 1). Consequently, the mean of efficacy index at 12 months following the last stage was 1.6 ± 0.6 (SD) (95% confidence interval [CI]: 1.3 – 1.8). The geometric mean of the safety index at 12 months after procedure completion was 2.6 (95% CI: 2.3 – 3.1). Both the efficacy and safety indexes were greater than 1, indicating that postoperative UDVA and CDVA exceeded the preoperative CDVA. Notably, all eyes gained one or more lines of CDVA, and no eye lost a single line of CDVA following treatment (Figure 1). In addition, the safety index was to be higher as the preoperative CDVA was to be worse (Spearman r = 0.948;* P* < 0.001), while the efficacy index was not statistically changed according to the preoperative CDVA (Spearman r = 0.169;* P* = 0.370) (Supplementary Figure (available here)).

**Figure 2:**
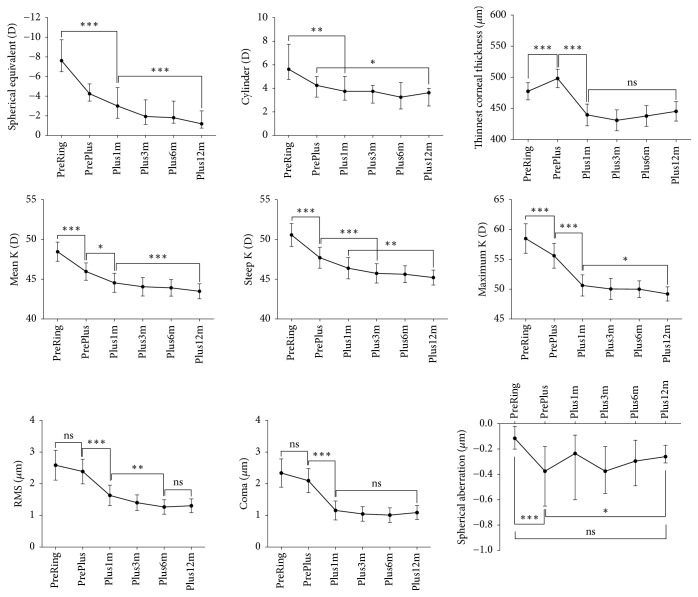
*Changes in various parameters before and after the two-staged multimodal surgery for keratoconus.* The error bars are the 95% confidence intervals of medians (spherical equivalent, cylinder, and spherical aberration) or means (the others). The spherical equivalent and the cylinder are measured by autorefraction. *∗∗∗*,* P* < 0.001; *∗∗*,* P* < 0.01; *∗*,* P* < 0.05; ns, not significant (Friedman test and Dunn's multiple comparisons test for spherical equivalent, cylinder, and spherical aberration; repeated ANOVA and Sidak's multiple comparisons test for corneal thickness, keratometric measurements, RMS, and coma). PreRing, before intracorneal ring segment implantation; PrePlus, before PRK-CXL (“Plus” surgery); Plus1m, 1 month after PRK-CXL; Plus3m, 3 months after PRK-CXL; Plus6m, 6 months after PRK-CXL; Plus12m, 12 months after PRK-CXL; K, keratometry; RMS, root mean square higher-order aberration.

The extent of myopia decreased after both ICRS implantation and PRK-CXL through at least 12 months after PRK-CXL treatment (Figure 2). The median spherical equivalent was – 1.2 D (95% CI: −2.5 D to -0.8 D) at 12 months after PRK-CXL. In addition, the mean K, steep K, and maximum K declined after both the initial ICRS implantation and the subsequent PRK-CXL through at least 12 months of follow-up after completion of both treatment stages (Figure 2). The magnitude of the cylinder began to be reduced at 1 month following completion of the protocol (Figure 2). The median cylinder at 12 months was 3.6 D (95% CI: 2.5 to 4.0 D), a statistically significant change (*P* < 0.001) from the median preoperative cylinder of 5.6 D (95% CI: 4.8 to 7.8 D) (Figure 2). Therefore, both ICRS and PRK-CXL additively contributed to the observed total drop in the magnitude of the cylinder.

With respect to corneal higher-order aberrations, the total HOA RMS was not changed by ICRS implantation but did decline by 6 months after PRK-CXL (Figure 2). Of the various types of HOAs, ICRS implantation had a neutral effect on coma and increased spherical aberrations, whereas PRK-CXL resulted in stable decreases in both coma and spherical aberration by 1 month and 12 months after PRK-CXL, respectively (Figure 2).

The minimum measured corneal thickness (CT_thinnest_) was increased somewhat after ICRS implantation and decreased, as expected, by PRK-CXL (Figure 2). After 1-month post-PRK-CXL treatment, the CT_thinnest_ remained stable without any significant later change (Figure 2).

A demarcation line in the corneal stroma (26 eyes, 87% of subjects) or mild to moderate superficial corneal haze (6 eyes, 20% of subjects) was observed at 12 months after PRK-CXL. There were no other serious complications such as a persistent corneal epithelial defect, ICRS protrusion, ICRS dislocation, deep corneal vascularization, or corneal infection [21, 22].

## 4. Discussion

The clinical outcomes of a two-stage approach to treating moderate keratoconus, consisting of ICRS implantation followed by PRK-CXL, were evaluated through one year of follow-up. The current surgery was particularly effective in enhancing CDVA and all patients improved their postoperative CDVA to 6/12 Snellen or better (Figure 1). In comparison to prior reports, the enrolled patients in this study on average had more advanced keratoconus (Table 1), yet they had better postoperative visual outcomes (Figure 1). This outcome is clinically noteworthy because this treatment option may afford patients with moderate keratoconus the opportunity to obtain more satisfactory vision with spectacle wear (Figure 1).

The treatment protocol of Al-Tuwairqi et al. did not appear to improve CDVA in their subjects, whereas the protocol reported by Lee et al. improved CDVA in their subjects, an effect similar to what we observed in our current study (Figure 4). Since both the Al-Tuwairqi and Lee protocols used Keraring for ICRS implantation, it is possible that the observed difference in results may be attributed to the use of topography-guided transepithelial PRK (TG-transPRK) and conventional CXL in the Al-Tuwairqi study in contrast to the corneal wavefront-guided transepithelial PRK (CWG-transPRK) and accelerated cross-linking (accCXL) approaches used by Lee and associates [9, 10]. The favorable results obtained in this study, which employed CWG-transPRK and accCXL but used the Intacs SK ring, were equal to or better than those obtained by Lee and colleagues. We speculate that use of CWG-transPRK may provide advantages in the accuracy of correction of highly aberrated corneas compared to the use of TG-transPRK in these eyes.

**Figure 3:**
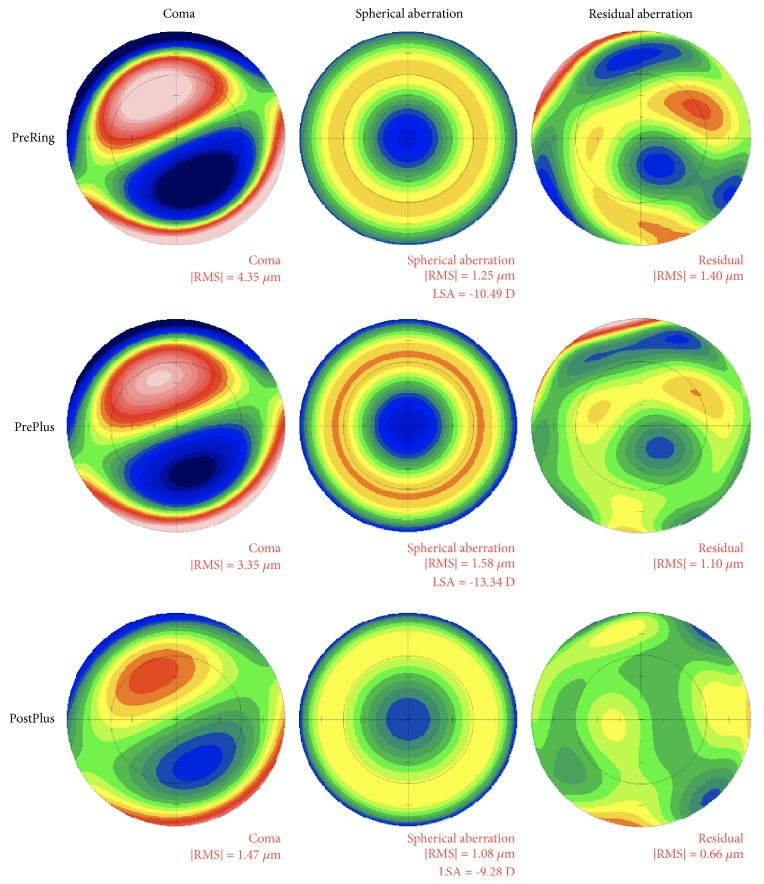
*Representative cases showing changes in higher-order aberrations.* Coma, spherical aberration, and residual aberration were clearly altered after PRK-CXL surgery (“Plus” surgery). Colors closer to blue-black have more negative coefficients (*μ*m); colors closer to pink have more positive values. Green indicates a zero value. PreRing, before intracorneal ring segment implantation; PrePlus, before PRK-CXL; PostPlus, after PRK-CXL.

In the current study, CDVA and UDVA were enhanced after both the initial stage of ICRS implantation and the subsequent stage of PRK-CXL (Figure 1). The Intacs SK reduced myopia by flattening the cornea and decreasing measured keratometry values (Figure 2). PRK-CXL addressed the residual myopia, astigmatism (lower-order aberrations), and high-order aberrations (Figures 2 and 3). Interestingly, the Intacs SK implantation did not in fact reduce HOAs (Figure 2). Indeed, additional negative spherical aberration developed after Intacs SK implantation (Figures 2 and 3). Reports from several groups of authors have confirmed that Intacs implantation improves UDVA, CDVA, and refractive error [4]. Other studies have found no significant change in corneal HOA after Intacs implantation [23, 24]. Interestingly, one study agreed with our finding that the Intacs induced negative spherical aberration [25], which we speculate may be caused by peripheral corneal flattening around the implant.

**Figure 4:**
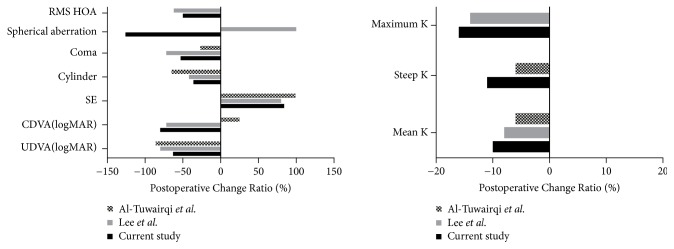
*Review and comparison of results by two-stage multimodal protocols, reported by Al-Tuwairqi et al. [9], Lee et al. [10], and the current study.* Postoperative change ratio is calculated as (postoperative value – preoperative value) ÷ preoperative absolute value x 100. RMS HOA, root mean square higher-order aberration; SE, spherical equivalent of refraction (current study, objective refraction; others, subjective refraction); CDVA, corrected distance visual acuity; UDVA, uncorrected distance visual acuity; K, keratometry.

In contrast to our findings, Lee and associates noted no significant change in corneal aberrations after Keraring implantation [10]. We speculate that the differing physical characteristics in Intacs SK versus Keraring segments may explain these observations. Notably, the Intacs SK had an internal diameter of 6 mm, which was greater than the 5 mm diameter of the Keraring implant. In addition, Intacs SK segments are thicker than Keraring and may have stronger flattening effect around the ICRS. A greater magnitude of flattening achieved over a larger optical zone after Intacs SK implantation might also explain the induction of negative spherical aberration as well as the greater reduction in K values we observed in our study (Figure 4). Notwithstanding, the induced negative spherical aberration after Intacs SK implantation did not seem to compromise CDVA and further improvement in CDVA was obtained after completion of PRK-CXL (Figures 1 and 2).

The protocols of Al-Tuwairqi and coauthors did not yield statistically significant reductions in coma (Figure 4) [9, 17]. They used the Schwind Corneal Wavefront Analyzer for topography-guided PRK [9, 26], which plans treatment solely on Placido ring-derived topographic measurements. Placido-based topography may yield significant test-to-test variation due to small variations in alignment with respect to the apex of the cone. Corneal tomography-based methods, such as Scheimpflug-based systems, are less subject to this error in keratoconic eyes [27]. In this study, we employed the Schwind Sirius, which uses both Placido disks and the Scheimpflug camera to generate a theoretically derived corneal wavefront [18]. We do note, however, that Lee and colleagues were able to achieve comparable reductions in coma with their Placido-based topography system as we observed with our protocol (Figure 4); this might be due to the milder severity of their patient cohort, better operator performance, or other factors.

After PRK-CXL was completed, stability in coma and corneal thickness was achieved by 1 month (Figure 2). However, UDVA, CDVA, refractive spherical equivalent, refractive cylinder, mean K, steep K, and maximum K continued to improve until 12 months (Figures 1 and 2). Therefore, a longer follow-up study may be required to fully evaluate the outcome of the two-stage protocol used in this study, since additional improvement of vision beyond 12 months is possible.

Interestingly, we observed that, after Intacs SK implantation, the measurements of the thinnest corneal point consistently increased by a modest but appreciable degree (Figure 2). This finding has been previously reported, but the reasons for this phenomenon are not clear. It has been speculated that ICRS implantation may lead to central corneal collagen crowding and stromal infolding [28]. This increase afforded the subjects in this study a greater margin of ablation depth available for PRK and/or a greater depth of cross-linking treatment without risking endothelial cell damage. Lee and associates did not observe a similar phenomenon of central corneal thickness increase after Keraring implantation [10]; the reasons for the differences between their study and ours are unknown, yet.

However, increase in the thinnest point could be important in the non-topography-guided vs. topography-guided PRK issue. Topography-guided PRK can raise a safety issue because it aims to normalize the cornea and a greater ablation depth occurs at the steepest and thinnest point of the cornea [29, 30]. Non-topography-guided PRK may be an option in cases of mild keratoconus with good CDVA to limit the ablation depth [29, 31]. However, topography-guided PRK can normalize the corneal shape, and it can be more effective in correcting HOA. Therefore, if the surgeon considers the laser ablation for the patient whose cornea is thick enough and still has irregular astigmatism remaining after ICRS implantation, topography-guided PRK would be more advantageous for the correction of HOA and an increase in CDVA. Nevertheless, in case of mildly irregular astigmatism with good CDVA after ICRS, non-topography-guided PRK may be used to ensure good vision and safety.

Lack of the control group and the small number of subjects were limitations to this study. To compensate for those limitations, we thoroughly compared our results with previous reports and carefully performed statistical analysis.

## 5. Conclusions

In conclusion, Intacs SK implantation followed by corneal wavefront-guided transepithelial photorefractive keratectomy combined with accelerated cross-linking in moderate keratoconus showed favorable results at 1 year, with improvements in best corrected and uncorrected visual acuity and no significant loss of best corrected visual acuity. These preliminary results improve upon the results of prior studies and suggest that this two-stage, multimodal approach may represent a promising option for the management of moderate keratoconus. Further study of this approach, ideally including a larger-scale, longer-term prospective randomized clinical trial, may be useful in validating these findings and optimizing treatment parameters.

## Figures and Tables

**Table 1:** Comparison of baseline patient characteristics in the three studies utilizing a two-stage surgical approach for the treatment of moderate keratoconus.

	Current study	Al-Tuwairqi* et al*.^a^	Lee *et al*.^b^
*Subject number (eyes)*	30	41	23

*Follow-up period*	1 year	1 year	6 months

*Surgical modality*			

ICRS type	Intacs SK	Keraring	Keraring
Excimer laser for transepithelial PRK	Schwind Amaris 1050	Schwind Amaris	Schwind Amaris 1050
Topographer type	Placido disk combined with Scheimpflug camera (Schwind Sirius)	Placido disk (Corneal Wavefront Analyzer, Schwind)	Placido disk (Keraton Scout, Optikon)
CXL	Accelerated (3 min at 30 mW/cm^2^)	Conventional (30 min at 3 mW/cm^2^)	Accelerated (6 min at 15 mW/cm^2^)

*Preoperative measurements, mean or median (95% confidence intervals)*			

UDVA (logMAR)	0.8^c^ (0.7 - 2)	0.74 (0.58 - 0.91)	0.85 (0.74 - 0.96)
CDVA (logMAR)	0.5 (0.4 - 0.6)	0.04 (0.00 - 0.08)	0.25 (0.18 - 0.32)

Spherical equivalent (D)	– 6.50^c,d^ (– 8.75 to – 5.00)	– 3.03^e^ (– 3.64 to – 2.42)	- 2.33^e^ (– 3.24 to – 1.42)
Cylinder (D)	6.32^d^ (5.17 - 7.46)	2.20^e^ (1.75 - 2.65)	1.83^e^ (1.27 - 2.39)

Steep K (D)	50.58 (49.15 - 52.00)	46.13 (45.49 - 46.77)	48.0 (46.5 - 49.5)
Mean K (D)	48.46 (47.25 - 49.67)	44.96 (44.40 - 45.52)	47.2 (45.9 - 48.5)
Maximum K (D)	58.5 (56.02 - 60.98)	NA	55.35 (53.10 - 57.60)

Thinnest corneal thickness (*μ*m)	478 (464 - 491)	501.87 (492.23 - 511.51)	463.9^f^ (451.4 - 476.4)

Coma aberration (*μ*m)	2.339 (1.891 - 2.787)	1.08 (0.88 - 1.28)	2.47 (2.06 – 2.88)
Spherical aberration (*μ*m)	– 0.115^c^ (– 0.2 to – 0.02)	NA	0.15 (– 0.09 to 0.39)
RMS HOA (*μ*m)	2.585 (2.117 - 3.054)	NA	2.87 (2.40 – 3.34)

^a^ [9].

^b^ [10].

^c^median; others are means.

^d^Subjective refraction was utilized for comparison purposes in this table; objective refraction data by autorefraction are presented in the text.

^e^Subjective refraction.

^f^Central corneal thickness.

ICRS, intrastromal corneal ring segment; PRK, photorefractive keratectomy; CXL, corneal collagen crosslinking; UDVA, uncorrected distance visual acuity; CDVA, corrected distance visual acuity; K, keratometry; RMS HOA, root mean square of higher-order aberrations; NA, not available.

## Data Availability

The clinical data used to support the findings of this study are available from the corresponding author upon request.

## Supplementary Materials

Supplementary Materials Supplementary Figure. Correlation between outcome indexes and preoperative corrected distance visual acuity (CDVA). The safety index is positively correlated with the CDVA (logMAR) while the efficacy index does not show correlations.
