# Supplementary figures and images for: One-Year Efficacy and Safety of Combined Photorefractive Keratectomy and Accelerated Corneal Collagen Cross-Linking after Intacs SK Intracorneal Ring Segment Implantation in Moderate Keratoconus
Source: Biomed Res Int. 2019 Jul 8;2019:7850216. doi: 10.1155/2019/7850216 (PMC6644267; doi:10.1155/2019/7850216)

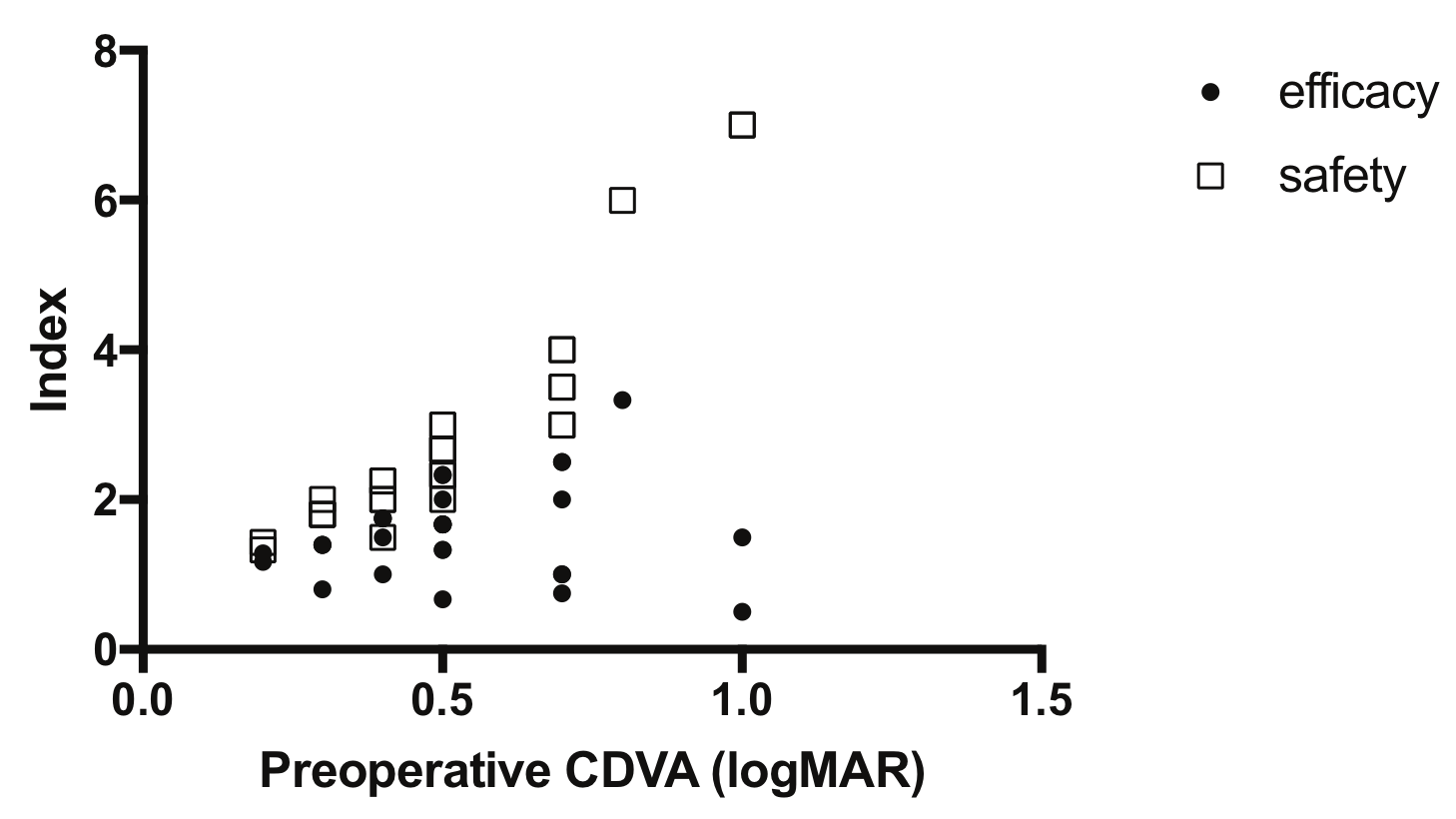

Supplement: Supplementary Materials — Supplementary Figure. Correlation between outcome indexes and preoperative corrected distance visual acuity (CDVA). The safety index is positively correlated with the CDVA (logMAR) while the efficacy index does not show correlations. [file 7850216.f1.tiff]
